# Editorial: Short and long-term treatment options in patients with acute coronary syndrome

**DOI:** 10.3389/fcvm.2025.1563882

**Published:** 2025-02-04

**Authors:** Antonio Landi, Albert Varga, Verena Veulemans, Andrea Milzi

**Affiliations:** ^1^ Cardiocentro Ticino Institute, Ente Ospedaliero Cantonale (EOC), Lugano, Switzerland; ^2^ Faculty of Biomedical Sciences, University of Italian Switzerland, Lugano, Switzerland; ^3^ Institute of Family Medicine, University of Szeged, Szeged, Hungary; ^4^ Department of Cardiology, Helios Heart Centre Siegburg, Siegburg, Germany

**Keywords:** acute coronary syndrome, coronary artery disease, secondary prevention, percutaneous coronary intervention, medical therapy

**Editorial on the Research Topic**
Short and long-term treatment options in patients with acute coronary syndrome

Coronary artery disease (CAD) and acute coronary syndromes (ACS) remain the leading causes of mortality in Western countries (1). In the last decades, technological advancements and optimization of pharmacological and interventional strategies led to a substantial reduction of ACS-associated morbidity and mortality (2). However, a not negligible proportion of post-ACS patients are still exposed to higher risks of fatal and non-fatal adverse events, including re-admission due to myocardial infarction (MI) or heart failure (HF), unplanned revascularization, stroke and bleeding.

Percutaneous coronary intervention (PCI) represents the revascularization modality of choice in ACS patients (3). The wide adoption of radial access (4, 5), newer-generation drug-eluting stents, intravascular imaging-guidance (6), optimized periprocedural antithrombotic treatments (7–9) and novel mechanical thrombectomy devices (10, 11) paved the way to the interventional treatment even in “complex, high-risk and indicated patients” (CHIP). In spite of these advancements, uncertainties exist both in the management of specific high-risk subjects, such as patients following out-of-hospital cardiac arrest (OHCA) or those with diabetes, and on the optimal medical therapy following ACS. The papers published in this research topic shed interesting insights on these crucial aspects.

The safety and effectiveness of immediate vs. delayed invasive coronary angiography (CAG) in patients without persistent ST-segment elevation after OHCA have been investigated in three recent randomized clinical trials (RCTs). In the TOMAHAWK trial, including 554 post-OHCA patients without ST-segment elevation, there were no significant differences in the primary endpoint of all-cause death at 30 days between the two strategies [hazard ratio [HR], 1.28; 95% confidence interval [CI], 1.00–1.63; *P* = 0.06] (12). The COACT (13) and EMERGE (14) trials yelded similar results at 90 and 180 days, respectively. Ahmed et al. summarize all available evidence on this by performing a systematic review and meta-analysis of observational studies (*n* = 9) and RCT (*n* = 7) encompassing a total of 4,737 OHCA patients without ST-segment elevation. The authors found that, compared with delayed or no CAG, early CAG was associated with lower long-term mortality [odd ratio (OR), 0.66; 95% CI, 0.51–0.85] and increased favorable cerebral performance at discharge (OR, 1.49; 95% CI, 1.09–2.03). However, this benefit mainly accrued from observational studies, while no benefit of immediate CAG was found when RCTs were separately appraised. Therefore, these results should be interpreted with caution due to different timeframes for early/delayed CAG (ranging from <2 to 6 h for early and from 6 h to 30 days for delayed CAG) and potential unmeasured confounders in observational studies.

Diabetes mellitus (DM) is associated with an impaired prognosis following ACS (15). In a large retrospective analysis from the FAST-MI program (*n* = 9,181 MI patients of whom 22% had DM), Bouisset et al. investigated the prognostic impact of DM on long-term survival. After propensity score matching, DM was associated *per se* with a 30% higher risk of mortality (HR, 1.30; 95% CI, 1.17–1.45; *p* < 0.001). As correctly pointed out by the authors, DM-associated microvascular damage and coronary microvascular dysfunction (not captured in the study dataset) could potentially explain this mortality excess.

Patients with ACS undergoing PCI require a treatment combination of different pharmacological agents to reduce the risk of adverse events. The pharmacological cornerstones include antithrombotic agents, lipid-lowering treatments, anti-RAAS (renin–angiotensin–aldosterone system), β-blockers and metabolic-acting agents (if clinically indicated). Current European Society of Cardiology (ESC) guidelines provide a framework for a tailored implementation of different treatment options (3). Among ACS patients without indication for oral anticoagulation, dual antiplatelet therapy (DAPT) consisting of aspirin and a P2Y_12_ inhibitor is recommended for 12 months unless there is high bleeding risk (3, 16). DAPT de-escalation strategies have repeteadly shown to reduce bleeding without ischemic harm (17–21) and are recommended with class IIa–IIb by the 2023 ESC guidelines on ACS (3). Current guidelines recommend to achieve an LDL-colesterol level of <1.4 mmol/L (<55 mg/dl) and to reduce LDL-colesterol by ≥50% from baseline (class of recommendation I, level of evidence A) (3). β -blockers are recommended in patients with left ventricular dysfunction [ejection fraction (EF) ≤40%], while RAAS inhibitors are recommended in ACS patients with HF symptoms, LVEF ≤40%, DM, hypertension, and/or chronic kidney disease (both with class of recommendation I, level of evidence A).

The current research topic provides evidence on the value of β-blockers and sodium–glucose cotransporter (SGLT)-2 inhibitors in ACS. In a retrospective analysis from the CCC-ACS project (*n* = 113,650 ACS patients), Zhang et al. investigated the prognostic impact of β-blockers in ACS patients with concomitant chronic obstructive pulmonary disease (COPD). Of the 1,084 patients with ACS and COPD, 540 patients received an early β-blocker therapy (within 24 h after hospital admission), whereas 544 did not. After adjustment, the authors found that early β-blocker treatment was associated with a 77% relative reduction in the risk of all-cause death (OR, 0.33; 95% CI, 0.12–0.92; *P* = 0.035) and a 37% relative reduction in the risk of HF (OR, 0.63; 95% CI, 0.41–0.94; *P* = 0.025). These data confirm the efficacy of β-blocker therapy in this subpopulation and dispel concerns on the potential effect of β -blocker induced bronchospasm in COPD patients. In another retrospective study including 465 diabetic patients with ACS complicated by acute HF, Rahhal et al. investigated the impact of SGLT-2 inhibitors on short and long-term clinical outcomes. The primary study endpoint was the composite of ACS, HF hospitalization or all-cause mortality at 1 and 12 months after discharge. SGLT-2 treatment was associated with lower risks of the primary composite endpoint at 1 (HR, 0.20; 95% CI, 0.04–0.94; *P* = 0.041) and 12 months (HR, 0.46; 95% CI, 0.22–0.99; *P* = 0.046), mainly due to lower risks of HF hospitalization. These studies lend support to the use of early β-blocker treatment in ACS patients with COPD and SGLT-2 inhibitors in diabetic patients with ACS complicated by acute HF. Further larger, randomized studies are needed to confirm these findings.

In conclusion, despite notable improvements, a not negligible proportion of ACS patients remains at higher risk of adverse events. Importantly, studies in this research topic outline potential strategies to mitigate this “residual risk” in ACS patients.
